# Description of new *Dryocoetes* (Coleoptera, Curculionidae, Scolytinae) speciesfrom Afghanistan and Northern India and redescription of *Scolytoplatypus kunala* Strohm

**DOI:** 10.3897/zookeys.56.525

**Published:** 2010-09-17

**Authors:** Michail Yu. Mandelshtam, Alexander V. Petrov

**Affiliations:** 1 Bolshoy prospect, building 76, apt. 53, St.Petersburg, 199026, Russia; 2 Department of Ecology and Forest Protection, Moscow State Forest University, Mytishchi-5, 141005 Moscow

**Keywords:** Curculionidae, Scolytinae, Dryocoetes, Scolytoplatypus, Afghanistan, Northern India, new species

## Abstract

A new bark beetle species, Dryocoetes brownei from Northern India and Afghanistan, is described as a new to science and redescription of Scolytoplatypus kunala Strohmeyer, 1908, previously known only from the female holotype, is provided.

## Introduction

One more new species of Scolytinae in the genus Dryocoetes was discovered during study of materials kept in Natural History Museum, London (NHML), and several more examples of this species were found in the O.N. Kabakov collection from Afghanistan.

Oriental Scolytoplatypus species were recently reviewed by Beaver and Gebhardt (2006). This paper gave new insights into the taxonomy of the genus and has helped researchers to describe new species from the region. However, treatment of Scolytoplatypus kunala Strohmeyer, 1908 in most papers, including the cited one, is doubtful or erroneous. Schedl (1973) considered Scolytoplatypus kunala to be a senior synonym of Scolytoplatypus darjeelingi Stebbing, 1914, but Beaver and Gebhardt (2006) treated Scolytoplatypus kunala as a junior synonym of Scolytoplatypus daimio Blandford, 1893 and Scolytoplatypus siomio Blandford, 1893, but as a distinct species from Scolytoplatypus darjeelingi. Importantly, all Scolytoplatypus species known from Himalaya, besides Scolytoplatypus kunala, were recorded only in Eastern Himalaya, whereas Scolytoplatypus kunala was discovered in Kashmir Province, Western Himalaya (Strohmeyer, 1908). We have found three males and two females of Scolytoplatypus species collected by O.N. Kabakov in Afghanistan in Russian collections. After comparison of these specimens with the female holotype of Scolytoplatypus kunala we consider these specimens from Afghanistan to be conspecific with Scolytoplatypus kunala and provide redescription of both male and female of this poorly known species. Scolytoplatypus kunala is reinstated here as separate good species. So far we extend the geographical range of Oriental Scolytoplatypus to the west and consider Scolytoplatypus kunala to be the most “western” Scolytoplatypus species from Asia.

## Systematics

### Dryocoetes brownei

Mandelshtam & Petrov sp. n.

urn:lsid:zoobank.org:act:6510C1A7-5D09-45D5-A994-2AD2CAE8522F

#### Description.

##### Male.

Holotype (NHML) body length 5.1 mm, width 2.0 mm, other specimens (four female paratypes) 4.8–5.0 mm in length. Body reddish brown, essentially as in Dryocoetes autographus Ratz. All five studied specimens appear to be mature, not teneral.

Frons is rather densely but not coarsely, uniformly punctured, with an enlarged puncture in center forming a small fovea. Frontal surface shining, without reticulation. Vertex more sparsely punctured and with smaller punctures. A median longitudinal black line (sulcus) on vertex probably indicates internal strengthening of the head. Frons with rather long, fairly sparse, yellowish hair-like setae, not forming tufts typical of females of Dryocoetes species. Hair-like setae significantly longer at lateral sides of pubescent frontal area. Epistoma with long dense yellowish setae directed downwards. Eyes rather large, emarginate anteriorly. Antennae typical for genus: funiculus 5-segmented and club obliquely truncate with recurved sutures on anterior face, and one suture near apex on posterior face.

Pronotum slightly longer than wide (1.9 vs. 1.8 mm); sides subparallel in basal three fourth and apex simply rounded, without teeth on anterior margin. Pronotal surface generally granulate, more coarsely anteriorly, punctured area very small and restricted to central portion of pronotal base around impunctate median line extending approximately one fifth of pronotal length only. Pronotal surface between granules shining, without any reticulation. Sides and anterior margin of pronotum with long, curved, yellow hair-like setae.

Scutellum large, flat, flush with elytral surface, of same reddish color as elytra.

**Figure 1.**
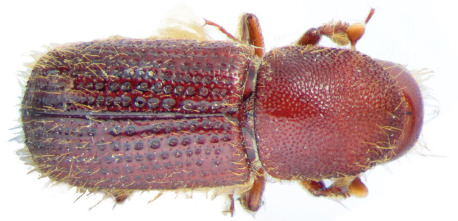
Dryocoetes brownei, HT, male, dorsal view.

Elytra slightly wider (2.0 vs. 1.9 mm) than pronotum, nearly cylindrical, only slightly widened towards apex, 3.2 mm long. Elytra extremely coarsely punctured on the disk, especially on striae 1–3 (Fig. 1) . Juxtasutural stria not impressed both on disk and on declivity, its punctures nearly as deep and as large as punctures of second stria. Interstriae in central part of disk narrow, 1.5–2.0 times narrower than striae, rather convex and shining, with minute punctures nearly 5 times smaller in diameter than strial punctures, in one irregular row on each interstria; minute tubercles present on interstriae on disk, but not on declivity.

Declivity slightly flattened, convex, not steep, dull. Ventrolateral sides of declivity not armed and lacking minute tubercles on declivital surface. First and second interstriae are widened at declivity. Strial punctures of first and second interstriae on declivity larger than punctures of other striae, but more than two times smaller than on disk. First and second striae slightly divergent towards elytral apex. Suture appearing only slightly convex on declivity because juxtasutural striae are not deepened. Elytra with rather long yellow interstrial hair-like setae, longer laterally and on the declivity; strial hair-like setae recumbent and approximately 5 times shorter that interstrial setae.

Legs typical for the genus, protibia with 5 socketed teeth on outer surface, meso- and metatibiae each with 5 spines on outer margin.

Underside of body reddish brown, abdomen covered with rather long setae on posterior margin of ventrites.

##### Female.

Similar to male, but the frons with a dense tuft of long yellow hair-like setae.

#### Etymology.

The new species is dedicated to the eminent British coleopterologist F.G. Browne, who first labeled the holotype as a possible new species.

#### Type material.

Holotype (male) is deposited in NHML. Labels of the holotype are as follows: Kashmir: Gulmarg. vi – 1931. Dr. M.Cameron. B.M. 931–452.// Dryocoetes sp.n. det. F.G.B. 1967 (in black ink, in F.G. Browne’s handwriting)// Possibly one of Beeson’s undescr. spp. – deobanos himalahinorum [sic!] (in black ink, in F.G. Browne’s handwriting). Paratypes (4 females) (including female allotype): Afghan[istan], Nurestan N.Waygal 2500 m., 10.VII.1972, Kabakov [leg.], 1 spec. in Zoological Institute, St.Petersburg (ZISP) and 3 spec. in A.V. Petrov private collection.

#### Diagnosis and comparison with other Indian Dryocoetes species.

A new bark-beetle is described from Northern India that differs from all other Indian species of the genus Dryocoetes by the exceptionally large punctures of the elytral discal striae and by the large size of the body.

In India, four species of genus Dryocoetes were previously reported to occur, namely Dryocoetes hewetti Stebbing, 1908, Dryocoetes himalayensis Strohmeyer, 1908, Dryocoetes quadrisulcatus Strohmeyer, 1908 and Dryocoetes indicus Stebbing, 1914. We were able to study all these four species in the Zoological Museum of Moscow University (ZMMU). The first of them has a spatulate antennal club, and extremely long declivital pubescence, and was correctly transferred to the genus Taphrorychus (Wood and Bright, 1992). Dryocoetes himalayensis and Dryocoetes quadrisulcatus differ from both Dryocoetes indicus and Dryocoetes brownei by the much smaller body size and by the strongly deepened first striae on the disk and the declivity. Although this feature is more evident in males of these species, it is also developed in the females. Dryocoetes indicus is also smaller in size (4.0 mm vs 5.1 mm) than Dryocoetes brownei, has equally punctured striae and interstriae on the disk, and minute but evident tubercles on the declivity.

### Scolytoplatypus kunala

Strohmeyer, 1908

#### Material examined:

Holotype (female): Kashmir, Pir Panjal, Rost [leg.]// Female sign // Type // Spongocerus kunala Strohm. Determ. Strohmeyer // Coll. Strohmeyer // Holotypus (on red paper). Holotype is deposited in Senckenberg Deutsches Entomologisches Iinstitut (SDEI). Additional material examined: 2 males, Afghan[istan], Nurestan, N. Waygal, 2750 m., 9.7.1972, Kabakov [leg.] (Zoological Museum of Moscow University and A.V.Petrov private collection); 1 more male and 2 females with labels: Afghan[istan] Nurestan N. Waygal 3500 m. 2.7.1972 Kabakov [leg.] (A.V. Petrov private collection).

#### Description.

##### Male.

**Figure 2.**
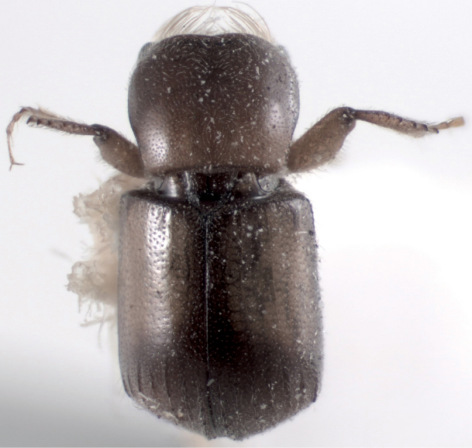
Scolytoplatypus kunala, male, dorsal view.

**Figure 3.**
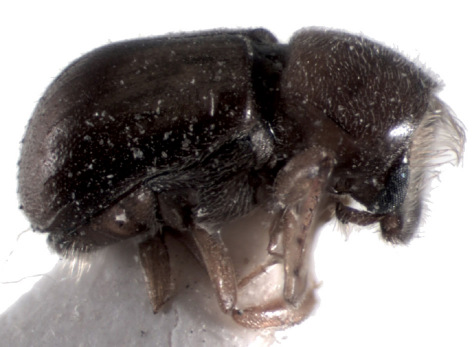
Scolytoplatypus kunala, male, lateral view.

Length 2.7 mm, body stout, 2.1 times as long as wide (Figs 2, 3). General colour pale brown, elytra with only slightly darkened suture, lateral margins and apices; each elytron with a large yellow spot extending from anterior margin up to two thirds of elytral length. Legs and antennae yellowish brown.

**Figure 4, 5.**
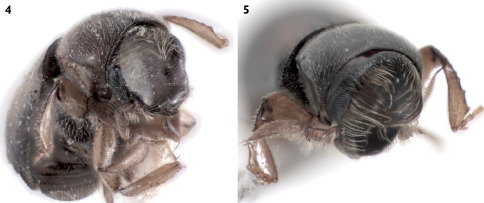
**4** Scolytoplatypus kunala, male, frontal vestiture. **5** Scolytoplatypus daimio, male, frontal vestiture.

Frons concave, uniformly shagreened, minutely, uniformly punctured. Vestiture of whole frontal surface consists of very fine erect and relatively long hairlike setae visible from lateral and dorsal view (not from frontal view). Upper and lateral edges of frons with long hairlike setae; those on upper part until middle of eyes very long and curved towards center of frons. Longest setae convergent in middle of frons, not extending beyond middle of frons; those on lateral edges of flattened lower frontal part become shorter ventrad and are not so curved (Fig. 4); frons is quite different from Scolytoplatypus daimio with frontal tufts of hairs attaining epistomal margin (Fig. 5).

Pronotum wide and short, 0.8 times as long as wide (1.9 mm vs 2.3 mm), widest in anterior third, vestiture of fine and short hair-like setae denser at anterior margin. Pronotal surface shining, minutely punctured, punctures very shallow and set 3–4 diameters apart from each other. Surface between punctures minutely reticulated. Posterior part of pronotum more lightly coloured than anterolateral angles. Lateral margins of pronotum sharply elevated and propleura strongly concave.

**Figure 6.**
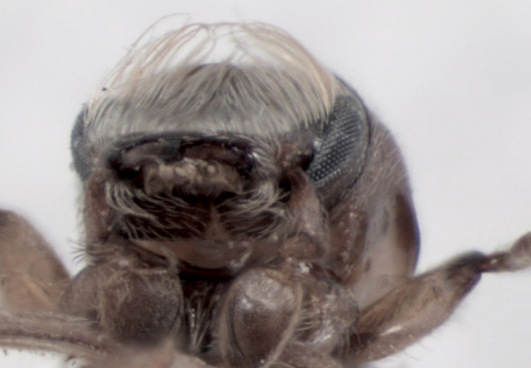
Scolytoplatypus kunala, male, prosternum.

**Figure 7.**
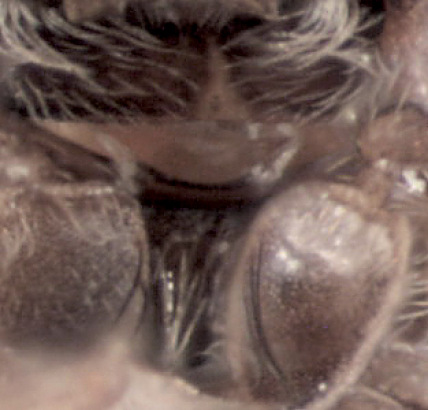
Scolytoplatypus kunala male prosternum organ, enlarged.

Prosternum weakly convex, with an obscure triangular elevation between procoxae, its indistinct pointed apex oriented backwards, and base of triangle forming anterior prosternal margin. This margin is armed with two divergent translucent processes set far apart (Figs 6, 7).

Scutellum small, triangular, flush with elytral surface.

Elytra 1.38 times as long as wide, 2.0 times as long as pronotum, clearly widened posteriorly. Elytral surface minutely punctured, shining, without signs of reticulation. Elytral punctures not organized in rows and interstriae invisible besides at declivity where all interstriae with exception of the first one are finely carinate. First declivital interstriae at declivity are broadly elevated, not carinate and bear 9–10 tubercles of median size, towards elytral apex evidently divergent. Carina on all other interstriae very low and devoid of tubercles. Elytral declivity convex, not impressed. Posterior dark carinate portion of elytra has yellowish and dense recumbent hair-like setae, anterior light portion of elytra is glabrous.

Underside of beetle is uniformly light yellow. Fourth and fifth abdominal sterna with abundant long yellowish hair-like setae protruding beyond abdominal apex.

Front femora without tooth above apex. Front tibiae with three widely set spines at lateral edge and with apical mucro. Tarsi long, third segment not bilobed, fifth segment as long as all previous combined.

##### Female.

**Figure 8.**
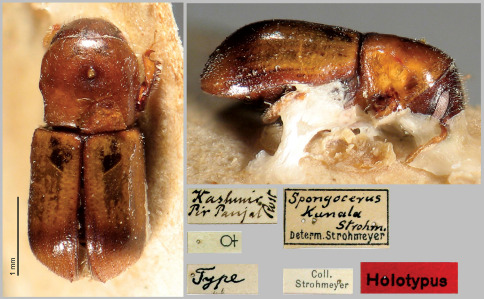
Scolytoplatypus kunala, Holotype, female.

Similar to male, but frons is not impressed and long pubescence at lateral and upper edges of frons is not developed (Fig. 8). Pronotum has a median mycangial pore in its centre.

Length 2.8 mm (HT – 2.96 mm), body stout, 2.15 times as long as wide. Body pale brown, elytral colour pattern essentially as in male, body surface faintly shining.

Head brown, darker compared to other body parts. Frons faintly convex, dull. Frontal surface gently shagreened. Lateral frontal parts near eyes faintly shining. Middle part of frons with a sickle-shaped faintly shining impression above mandibles, upper part of impression near center of the frons with two symmetrically placed small tubercles that are seen with difficulty. Slightly elevated dark median line continues from vertex down to epistomal impression. Frontal surface covered by shallow round punctures. Median part of frons covered by sparse, short erect hair-like setae, with somewhat higher density in middle of frons. Short recumbent hair-like setae are concentrated around eyes. Vertex is covered with round shallow punctures, glabrous. Antennae brown with sand-coloured triangular flat club.

Pronotum subquadrate, surface faintly shining, covered by microscopic round punctures, with faintly developed flattened tubercles laterally from pronotum center. Slightly anteriorly to pronotum middle a well-developed mycangial pore with a bunch of pale fine hair-like setae is developed. Posterior part of pronotum up to the central pore is covered by pale microscopic hair-like setae; anteriorly to the pore, pronotum with more long pale erect hair-like setae. Lateral pronotal margin as in all Scolytoplatypus is acute. Prothorax has an impression for protibiae. Intercoxal piece flat, its lateral margins near apex with bunches of long golden hair-like setae.

Scutellum as in male.

Elytra light brown, faintly shining. Elytral base 1.2 times as wide as pronotum base. Maximal elytral width at beginning of declivity. Declivital margins not evenly rounded toward elytral apex, elytral apex somewhat angular. Dorsum of declivity evenly arched towards apex. Elytra 1.4 times as long as wide. Elytral surface with circular punctures. Strial punctures not deepened and of similar size to interstrial punctures, so that striae are obscure, not clearly seen. Interstriae slightly elevated on declivity, first interstriae with minute tubercles, seen with difficulty. Elytral surface covered with microscopic light hair-like setae.

Metasternum light brown, covered by short pale hair-like setae. Metacoxal cavities with clearly marked raised margin.

Abdomen light brown. Sternites covered by minute shallow round punctures and pale hair-like setae; these setae clearly longer on 3-rd, 4-th and 5-th sternites than on 1-st and 2-nd sternites.

Legs light yellow. Denticles on outer lateral protibial surface rather strong in the specimen described, but probably intraspecifically variable.

#### Diagnosis.

In habitus the species resembles specimens of Scolytoplatypus daimio, but smaller in size and has another color pattern on elytra (with only poorly developed light dark pattern). Frontal pubescence essentially as in Scolytoplatypus zahradniki Knížek, 2008 or Scolytoplatypus tycon Blandford, 1893. Rather short frontal vestiture easily distinguishes the male Scolytoplatypus kunala from Scolytoplatypus daimio and Scolytoplatypus darjeelingi males in which the longest setae extend in a brush from the upper frontal parts up to the epistoma (Fig. 5). Based on the habitus and details of the male prosternum morphology, Scolytoplatypus kunala is most closely related to Scolytoplatypus daimio and Scolytoplatypus darjeelingi, differing from both species in the presence of rather short frontal vestiture in the male. The last feature is present not only in Scolytoplatypus kunala but also in Scolytoplatypus tycon, hovewer in the latter, the male prosternum is differently developed and the body is much stouter. The pale area of the elytra is surrounded only by slightly darker zone formed by brown elytral apices and elytral margins, not black as in Scolytoplatypus darjeelingi Strohmeyer, 1914. All other Oriental species can be easily distinguished by the features given in the modified key of Beaver and Gebhardt (2006) (see below). In the recently described Scolytoplatypus zahradniki Knížek, 2008 with similar frontal pubescence the male prosternum does possess two processes anteriorly, but they are closely set and not wide apart as in Scolytoplatypus kunala. A similar prosternum to Scolytoplatypus kunala is seen in Scolytoplatypus daimio, but not in Scolytoplatypus zahradniki, which possesses closely set translucent processes at anterior margin of pronotum and a distinct type of pronotal elevation with a sharp keel protruding forwards and backwards. The Scolytoplatypus zahradniki males can also be distinguished by the clearly carinate elytral interstriae which are only slightly elevated on the Scolytoplatypus kunala declivity (Knížek 2008). The female of Scolytoplatypus kunala can be distinguished from the females of Scolytoplatypus daimio and Scolytoplatypus zahradniki by the smaller body size and by presence of declivital tubercles only on the 1 – st elytral interstria, by very faint striae on the apical part of the elytral declivity.

Unfortunately, the recently published key to Indian Scolytoplatypus by Maiti and Saha (2009) ignores the paper by Beaver and Gebhardt (2006) and uses quite another set of features to distinguish species. Importantly, it does not use the male prosternum structure to provide principal features to identify species groups. Three new species were described as new by Maiti and Saha (2008), namely Scolytoplatypus gardneri, Scolytoplatypus lopchuensis and Scolytoplatypus samsinghensis; however use of another set of features to distinguish species does not allow the inclusion of these three species into the key by Beaver and Gebhardt (2006). Scolytoplatypus samsinghensis differs from Scolytoplatypus kunala by the much larger size (3.75–3.80 mm versus 2.70–3.00 mm), by the uniformly dark reddish brown to blackish brown body color, by the presence of conspicuous interstrial ridges on the elytral disk both in males and in females and also by the very short frontal pubescence both in male and in female. Besides, sparse long, erect declivital hair-like setae present in Scolytoplatypus samsinghensis male are absent in Scolytoplatypus kunala. Both Scolytoplatypus lopchuensis and Scolytoplatypus gardneri are slightly smaller in size compared to Scolytoplatypus kunala (2.55–2.60 mm vs. 2.70- 3.00 mm). The female of Scolytoplatypus lopchuensis differs from that of Scolytoplatypus kunala by the absence of the mycetangium pore in the centre of the pronotum, and the female of Scolytoplatypus gardneri is unknown. Males of both species, Scolytoplatypus lopchuensis and Scolytoplatypus gardneri, are different from Scolytoplatypus kunala in having even frontal vestiture without a fringe of hair-like setae on the upper frontal rim with their apices converging towards the centre of the frons. Scolytoplatypus kunala is very closely related to Scolytoplatypus darjeelingi Stebbing sensu Maiti and Saha 2009; however, in contrast to Maiti and Saha’s description, Scolytoplatypus darjeelingi has very long tufts of hair-like setae originating from the upper part of the frons (Stebbing 1914, Beaver and Gebhardt 2006) and extending to the epistoma near the mandibles.

#### Discussion.

The key to Oriental Scolytoplatypus species males by Beaver and Gebhardt (2006) can be easily modified to include both redescribed Scolytoplatypus kunala and recently described Scolytoplatypus zahradniki.

##### The key to males will be changed from couplet 19

**Table:** 

19.	Prosternum with a pair of widely separated, translucent, divergent anteriorly processes. Rows of punctures on elytral dorsum not impressed, usually indistinct	19A
–	Prosternum with a pair of closely set translucent processes, with an asymmetrical translucent process, or without translucent processes	19B
19A.	Frons with a rather sparse fringe of hair-like setae on each side curving inwardly, convergent to the center of frons but not extending to lower half of frons. 2.7 mm	Scolytoplatypus kunala Strohmeyer
–	The incurved brushes of hair-like setae denser and longer, extending beyond middle of frons and usually attaining epistomal margin	22 (species Scolytoplatypus shogun, Scolytoplatypus daimio and Scolytoplatypus darjeelingi)
19B.	Prosternum raised in middle in a triangle, the apex anterior or posterior	20
–	Prosternum flat or weakly convex, not raised in a triangle. Prosternum without translucent processes at the anterior margin. 3.5–4.5 mm	Scolytoplatypus tycon Blandford
20.	Apex of prosternal triangle posterior, anterior margin projecting in two rounded lobes, slightly asymmetrical, and with a translucent process on the right side only. 2.8–3.0 mm long	Scolytoplatypus ruficauda Eggers
–	Apex of prosternal triangle anterior, with two symmetrical, divergent, triangular, closely set translucent processes	21
21.	Posterior margin of raised prosternal triangle has a sharp elevation directed posteriorly in the center, apex of prosternal triangle anterior without a pointed tubercle directed downwards. 2.6–3.3 mm	Scolytoplatypus zahradniki Knížek
–	Apex of prosternal triangle anterior with a single pointed tubercle directed downwards. 3.1–3.3 mm long	Scolytoplatypus blandfordi Gebhardt

The key to Oriental Scolytoplatypus species females by Beaver and Gebhardt (2006) can also be modified to include the redescribed species, Scolytoplatypus daimio, and recently described Scolytoplatypus zahradniki Knížek.

##### The key to females will be changed from couplet 15

**Table:** 

15.	Basal angles of pronotum triangularly produced laterally, acute apically (species Scolytoplatypus mikado, Scolytoplatypus raja)	16
–	Basal angles of pronotum not strongly produced laterally, approximately rectangular	17
17.	Elytral interstriae carinate in the posterior two thirds of elytra 2.8–3.5 mm	Scolytoplatypus zahradniki Knížek
–	Elytral interstria not carinate, with rows of tubercles or striae weakly impressed before declivity or not impressed at all; if carinate than size less than 2.8 mm	17A
17A.	Elytral striae weakly impressed before declivity; elytral disc with fine hair-like setae on both striae and interstriae. Body length 3.8–4.5 mm	Scolytoplatypus tycon Blandford
–	Elytral striae not impressed before declivity, or if impressed, then length less than 2 mm1818.	Elytral declivity with dense vestiture of long, yellowish hair-like setae 19 (species Scolytoplatypus blandfordi, Scolytoplatypus darjeelingi, Scolytoplatypus pubescens)
–	Elytral vestiture of very short hair-like setae or elytra glabrous	21
21.	More elongate species, the elytra 1.7–1.9 times as long as pronotum	22 (species Scolytoplatypus shogun, Scolytoplatypus daimio)
–	Less elongate species, the elytra at most 1.5 times as long as pronotum	23
23.	Apex of interstriae 2 with a small acutely pointed tooth	24 (species Scolytoplatypus carinatus, Scolytoplatypus nitidus)
–	Apex of interstriae 2 without a tooth	24A
24A.	Larger species, body length 2.8–3.0 mm. Interstriae at the declivity with minute tubercles, clearly seen only at the first interstriae, obscure and seen with difficulty at all other interstriae	Scolytoplatypus kunala Strohmeyer
–	Smaller species 1.2–1.8 mm long	25 (species Scolytoplatypus nanus, Scolytoplatypus minimus, Scolytoplatypus pusillus)

## Supplementary Material

XML Treatment for Dryocoetes brownei

XML Treatment for Scolytoplatypus kunala
